# Norm values and psychometric properties for the German health regulatory focus scale – results of a representative survey

**DOI:** 10.1186/s12874-020-00927-x

**Published:** 2020-03-02

**Authors:** Bjarne Schmalbach, Markus Zenger, Elmar Brähler, Katja Petrowski

**Affiliations:** 1 Department of Psychosomatic Medicine and Psychotherapy, University Medical Center of the Johannes Gutenberg University Mainz, Untere Zahlbacher Str. 8, 55131 Mainz, Germany; 2 Faculty of Applied Human Studies, University of Applied Sciences Magdeburg-Stendal, Osterburger Str. 25, 39576 Stendal, Germany; 3 Integrated Research and Treatment Center AdiposityDiseases - Behavioral Medicine, Psychosomatic Medicine and Psychotherapy, University of Leipzig Medical Center, Philipp-Rosenthal-Straße 27, 04103 Leipzig, Germany; 4 Medical Psychology & Medical Sociology, University Medical Center of the Johannes Gutenberg University Mainz, Saarstraße 21, 55099 Mainz, Germany

**Keywords:** Regulatory focus, Health, Short scale, Psychometric properties, Norm values

## Abstract

**Background:**

The health regulatory focus is an application of Higgins’ regulatory focus theory to a health-specific context. It explains individual differences in health motivation, strategies, and behavior. Previous research found the Health Regulatory Focus Scale (HRFS) to be a reliable and valid measure for the construct. However, an evaluation of the HRFS in a representative sample has not been performed as of yet. Neither are there any normative values available.

**Methods:**

We collected a representative sample from the German general population to perform a confirmatory factor analysis, an analysis of measurement invariance, and to calculate norm values.

**Results:**

A two-factor model evinced good model fit with a good reliability for the two subscales. We found evidence for strict invariance across gender groups and partial strict invariance across age groups. In addition, we are presenting normative values for the general population.

**Conclusions:**

The findings of the present study are in line with previous research in confirming the HRFS as a valid and reliable tool suitable for the assessment of the health regulatory focus. The reported normative values allow for comparisons of individuals with their respective sociodemographic group.

## Background

The World Health Organization (WHO) defines health as “complete physical, mental, and social well-being and not merely the absence of disease or infirmity” [1; p. 1]. Hereby, the avoidance of risks as well as the effort toward general wellness is of importance. In order to ensure the efficacy of health interventions, the target population must be specified as accurately as possible [2] and the interventions modified to the specific needs [3] Therefore, individual differences concerning health behavior, needs, and strategies used to improve one’s health need to be taken into account.

To allow for an efficient assessment of individual differences in health motivation and behavior, Gomez and colleagues [4] developed the Health Regulatory Focus Scale (HRFS) evaluating the health-specific regulatory focus. The questionnaire assesses health promotion as well as health prevention. It is based on the regulatory focus theory which deals with gain- and loss-orientation in a general context [5] and has been adapted from general constructs to fit into the health context [4]. Based on previous research [6, 7], health promotion items focus on seeking and seizing opportunities to improve one’s health while health prevention items capture the attempts to avert dangers to one’s health.

Correlations between health prevention focus and health promotion focus vary between *r* = 0.16 and *r* = 0.57 [4]. In terms of convergent and discriminant validity, health promotion focus shows moderate correlations with optimism, positive self-evaluations, and approach motivation [8–10]. In contrast, health prevention focus is associated with neuroticism, negative self-evaluations, motivational inhibition, and impaired mental health [8, 11]. Finally, health promotion predicts health outcomes positively, while health prevention is associated negatively [12, 13].

For the German HRFS, reliability and item characteristics were found to be satisfactory [8]. Based on confirmatory factor analysis (CFA), a two-factor solution fits best for the HRFS. However, measurement invariance for gender and age was unclear due to Heywood cases. Furthermore, the sample was collected online and made up mainly of young female participants. Thus, it cannot be considered representative of the general population. Euhus et al. [9] addressed these concerns to some extent, but did not analyze a representative sample either.

In order to use and interpret individual results of the HRFS, normative values of a representative sample of the general population must be available. Therefore, the purpose of the present study is twofold. First, the aim is to build upon Schmalbach and colleagues [8] and find additional evidence for the psychometric qualities of the HRFS. Second, we seek to establish norm values for the HRFS to allow for the evaluation of individuals based on representative data from the German population.

## Method

### Participants

**Fig. 1:**
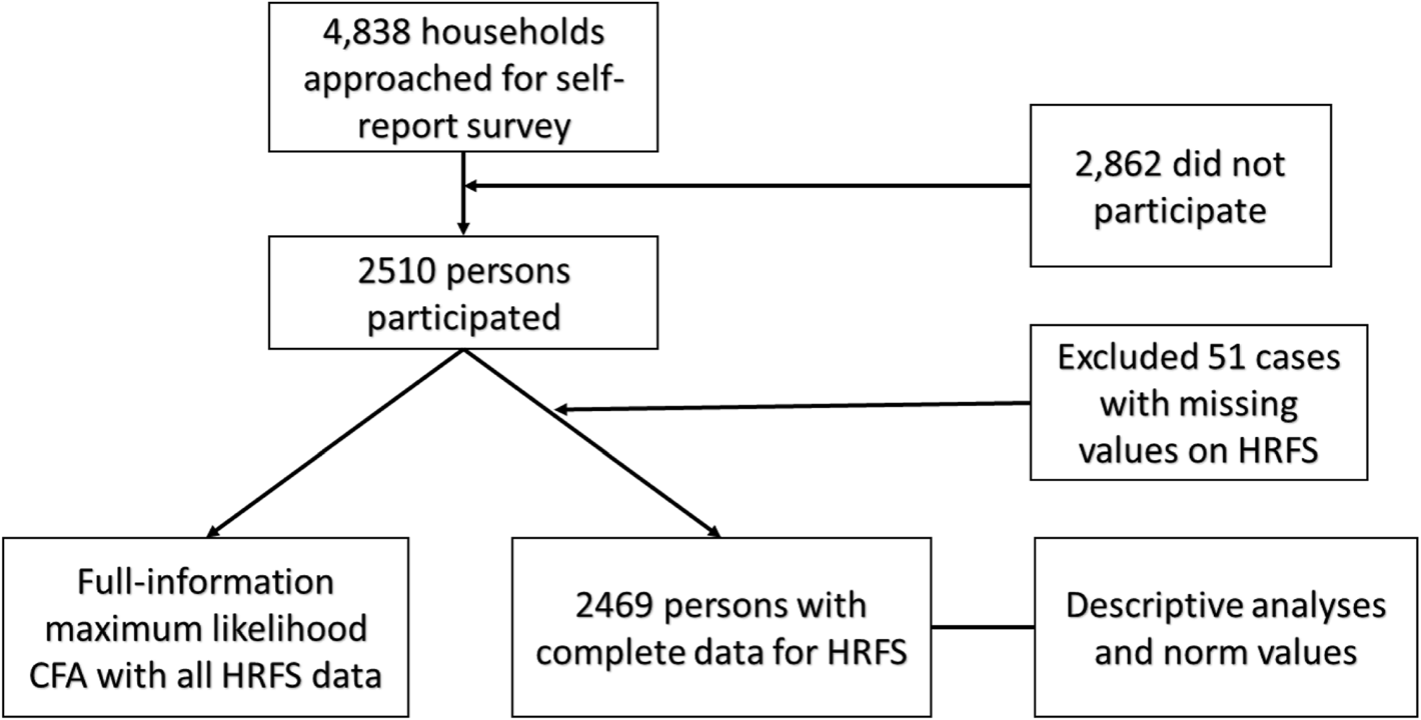
Sample flow chart

The full study sample consisted of 2510 participants. Of those, 2469 responded to all HRFS items and 40 responded to at least one of the items so that we were able to use their data in the factor analysis (see analysis section for more details). For descriptive statistics, comparisons, and norm values, only participants with complete data were included. Figure 1 illustrates the sample flow. A detailed sample description is reported in the Results section.

### Material

Gomez and colleagues [4] developed the HRFS to measure the promotion and prevention focus in health-specific contexts. Schmalbach and colleagues [8] translated the scale using a back-translation process and adapted it based on psychometric considerations. The German version has five items measuring the health promotion focus and two items for the health prevention focus. Response options are presented on a 7-point-scale ranging from (1) “strongly disagree” to (7) “strongly agree”. Taking the average of the items in question yields the respective scale scores for health promotion and health prevention. Apart from the HRFS, the participants gave their sociodemographic information.

In addition, we applied the Patient Health Questionnaire-4 (PHQ-4 [14]) to further test convergent validity. It is a screening instrument for anxiety and depression. As such it is one of the most widely-applied tools for the assessment of mental health. Respondents rate the frequency of symptoms of depression and anxiety they have experienced using two items each on a four-point scale ranging from “Not at all” to “Nearly every day”. Two subscale scores and a total score are calculated by adding up the item scores, ω_Anxiety_ = .798 [.780; .817], ω_Depression_ = .807 [.801; .824], ω_Total_ = .879 [.865; .892].

### Procedure

A representative sample of the general population of Germany was collected in 2016 by a demography consulting company (USUMA, Berlin). Per random-route procedure, households in all regions of Germany (based on electoral districts) were selected. Among the 4838 selected addresses, 2324 households (48%) could not be interviewed (e.g., illness, on holiday, refusal, unavailability) and 4 interviews (0.1%) were not analyzable (see Fig. 1). Within each household, the interviewee was selected based on the Kish selection grid. A trained professional then conducted the interviews with the consenting participants (oral consent in accordance with German law). The resulting sample was representative for the German community regarding age, gender, and education, as evidenced by comparisons with the Federal Statistical Office [15].

### Analysis

We conducted the statistical analysis using R and the packages lavaan and semTools [16, 17]. To enable the inclusion of participants with partially missing data and in order to address the slightly skewed and kurtotic distribution of some items, we used the robust full-information maximum likelihood estimation method with Yuan-Bentler’s scaled χ^2^ [18–21]. To evaluate goodness-of-fit, we utilized popular fit indices with commonly recommended cut-off criteria for good fit [22–24]: The χ^2^-test which should ideally not be significant; χ^2^ divided by degrees of freedom (CMIN/DF), which should be smaller than 3; the Comparative Fit Index (CFI), and the Tucker-Lewis Index (TLI), which should be larger than .95, the Root Mean Square Error of Approximation (RMSEA) and its 90% confidence interval, and the Standardized Root Means Square Residual (SRMR), which should be smaller than .08.

We tested measurement invariance across age and gender using the procedure recommended by previous research [25, 26]: We compared increasingly constrained models in a stepwise fashion to establish increasingly strict levels of invariance. Firstly, we tested metric (or weak) invariance by comparing the unconstrained model with a model that constrains factor loadings to be equal across groups. Secondly, we tested scalar (or strong) invariance by comparing the metric model to one that additionally constrains item intercepts to be equal. Finally, we tested strict invariance by comparing the scalar model to a model that also constrains residuals to be equal across tested groups. As recommended by Milfont and Fischer [26], we evaluated model comparisons using the χ^2^-test as well as differences in CFI and gamma hat (GH [27];). χ^2^ should ideally not be significant, and ΔCFI and ΔGH should not be larger than .01 between models. As per recommendations by Trizano-Hermosilla and Alvarado [28], we report McDonald’s ω [29] as a measure of internal consistency.

## Results

We report a detailed description of the sample in Table 1. Participants were on average 48.36 years old (*SD* = 18.22). The mean monthly net household income was 2592.86 euros (*SD* = 1485.10 euros). It correlated very weakly with health promotion, *r*(2,362) = .026, *p* = .206, and health prevention focus, *r*(2,362) = −.078, *p* < .001. The sample can be considered as representative of the German population.

**Table 1:** Sociodemographic characteristics of the sample along with comparisons in HRFS

	N	%	HRFS Promotion	HRFS Prevention
Gender			*F*(1, 2464) = 49.28, *p* < .001, η^2^_*p*_ = .020	*F*(1, 2464) = 19.94, *p* < .001, η^2^_*p*_ = .008
Female	1339	53.3	4.41^a^ (1.40)	3.42^a^ (1.61)
Male	1171	46.7	4.01^b^ (1.49)	3.13^b^ (1.65)
Age groups (years)			*F*(2, 2463) = 7.32, *p* = .001, η^2^_*p*_ = .006	*F*(2, 2463) = 24.97, *p* < .001, η^2^_*p*_ = .020
≤ 39	873	34.8	4.07^a^ (1.47)	2.97^a^ (1.62)
40–59	893	35.6	4.30^b^ (1.45)	3.39^b^ (1.61)
≥ 60	744	29.6	4.32^b^ (1.43)	3.52^b^ (1.62)
Education			*F*(3, 2453) = 7.80, *p* < .001, η^2^_*p*_ = .009	*F*(3, 2453) = 18.48, *p* < .001, η^2^_*p*_ = .022
≥ 9 years	809	32.2	4.21^a^ (1.41)	3.55^a^ (1.64)
10 years	1071	42.7	4.30^a^ (1.50)	3.26^b^ (1.61)
≤ 11 years	543	21.6	4.22^a^ (1.36)	3.06^c^ (1.58)
Pupil/Student	78	3.1	3.51^b^ (1.73)	2.38^d^ (1.75)
Missing	9	0.4		
Family			*F*(5, 2451) = 3.78, *p* = .002, η^2^_*p*_ = .008	*F*(5, 2451) = 4.71, *p* < .001, η^2^_*p*_ = .010
Married	1093	43.5	4.25^a^ (1.42)	3.27^a^ (1.63)
Separated	53	2.1	4.36^a^ (1.57)	3.55^a^ (1.58)
Single	614	24.5	4.02^b^ (1.48)	3.11^a^ (1.67)
Divorced	347	13.8	4.34^a^ (1.49)	3.32^a^ (1.53)
Widowed	221	8.8	4.38^a^ (1.45)	3.70^b^ (1.68)
Committed Relationship	173	6.9	4.37^a^ (1.38)	3.30^a^ (1.61)
Missing	9	0.4		
Employment			*F*(3, 2443) = 6.95, *p* < .001, η^2^_*p*_ = .008	*F*(3, 2443) = 16.78, *p* < .001, η^2^_*p*_ = .020
Working	1415	56.4	4.23^a^ (1.45)	3.18^a^ (1.60)
Retired	638	25.4	4.36^a^ (1.43)	3.60^b^ (1.64)
School/Apprenticeship	203	8.1	3.83^b^ (1.53)	2.77^c^ (1.67)
Not working	235	9.4	4.21^a^ (1.44)	3.45^b^ (1.61)
Missing	19	0.8		

Means (Standard Deviations) of the HRFS subscales for each sociodemographic group, along with ANOVAs. Differing superscripts denote significant differences between sociodemographic groups

The Shapiro-Wilk test for normal distribution was highly significant, *p* < .001, for all items and both scales. Considering our sample size, however, this is not surprising. Instead, Kim [30] recommends the analysis of skewness and kurtosis of the distributions. Absolute values for skewness should be smaller than 2 and excessive kurtosis smaller than 4, which was the case with our items and scales. Reliability for the promotion scale is ω = .916 [910, 923] while the prevention scale had a reliability of ω = .813 [.805; .821]. Further statistics are reported in Table 2.

**Table 2:** Item and scale characteristics

	*M*	*SD*	γ_1_	γ_2_	*r*_it_	λ
HRFS Item 1	4.44	1.75	−.30	−.80	.71	.74
HRFS Item 2	4.32	1.66	−.24	−.68	.81	.83
HRFS Item 3	3.94	1.67	.02	−.72	.77	.82
HRFS Item 4	4.24	1.64	−.17	−.69	.82	.87
HRFS Item 5	4.20	1.68	−.12	−.78	.83	.88
HRFS Promotion scale	4.23	1.45	−.19	−.48		
HRFS Item 6	3.44	1.84	.27	−1.03	.68	.85
HRFS Item 8	3.13	1.72	.42	−.78	.68	.81
HRFS Prevention scale	3.29	1.63	.30	−.80		

*γ*_*1*_ skewness, *γ*_*2*_ excessive kurtosis, *r*_*it*_ corrected item-total-correlation, *λ* standardized factor loadings

Overall, the model fits reasonably well, χ^2^(13) = 229.53, *p* < .001, χ^2^/*df* = 17.66, *CFI* = .968, *RMSEA* = .081 [.074; .089], *TLI* = .949, *SRMR* = .022, confirming the findings by Schmalbach and colleagues [8]. Specifically, the χ^2^-test and χ^2^ divided by degrees of freedom indicate bad fit, which is not surprising given χ^2^’s sensitivity to sample size [31]. The fit indices, on the other hand, reveal an acceptable model fit. CFI and SRMR present evidence for good fit, whereas RMSEA and TLI are barely acceptable.

We found clear evidence for strict invariance across gender groups, as evidenced by small differences in CFI and GH (see Table 3). The χ^2^-test was significant in one of the three comparisons. For age groups, we found evidence for metric invariance. By freeing the intercepts of items 1, 2, and 4 to vary between groups, we were able to show partial strict invariance. In addition, we tested for invariance across education, family, and employment status, utilizing these subgroups (from Table 1) that had at least 200 observations. For education and family, the evidence suggest strict invariance, whereas for employment status there were some differences with regard to item intercepts. However, partial strict invariance could be established for this grouping variable. Overall, the HRFS appears to be highly invariant across sociodemographic variables – with some minor limitations with regard to age and employment.

**Table 3:** Analysis of measurement invariance

Model	χ^2^(*df*)	Δχ^2^	*p*	*CFI*	Δ*CFI*	*GH*	Δ*GH*
Gender multi-group analysis (female / male)							
Configural invariance	248.92 (26)			.967		.951	
Metric invariance	265.85 (31)	16.93	.342	.965	.002	.949	.002
Scalar invariance	278.93 (36)	13.08	< .001	.964	.001	.947	.002
Strict invariance	269.00 (43)	9.93	.248	.967	.003	.951	.004
Age multi-group analysis (≤39, 40–59, ≥60)							
Configural invariance	250.44 (39)			.970		.932	
Metric invariance	274.31 (49)	23.87	.008	.968	.002	.927	.005
Scalar invariance	409.34 (59)	135.03	< .001	.950	.018	.891	.036
Partial scalar invariance ^a^	298.23 (53)	23.92	< .001	.965	.003	.922	.005
Partial strict invariance ^a^	294.17 (67)	4.06	.852	.967	.002	.927	.005
Education multi-group analysis (≥ 9 years, 10 years, ≤ 11 years)							
Configural invariance	249.921 (39)			.973		.976	
Metric invariance	272.582 (49)	22.661	.012	.973	.000	.974	.002
Scalar invariance	335.692 (59)	63.110	< .001	.967	.006	.968	.006
Strict invariance	332.419 (73)	3.273	.998	.967	.000	.970	.002
Family status multi-group analysis (married, single, divorced, widowed)							
Configural invariance	241.489 (52)			.973		.976	
Metric invariance	288.811 (67)	47.322	< .001	.972	.001	.973	.003
Scalar invariance	378.669 (82)	89.858	< .001	.965	.007	.964	.009
Strict invariance	415.905 (103)	37.236	.016	.960	.005	.962	.002
Employment status multi-group analysis (working, retired, school/apprenticeship, not working)							
Configural invariance	273.037 (52)			.974		.975	
Metric invariance	306.536 (67)	33.499	.004	.974	.000	.973	.002
Scalar invariance	457.330 (82)	150.794	< .001	.960	.014	.959	.014
Partial scalar invariance^b^	402.601 (79)	96.065	< .001	.965	.009	.964	.009
Partial strict invariance^b^	422.636 (100)	20.035	.331	.963	.002	.964	.000

^a^Intercepts of Items 1, 2, and 4 were freed to vary between groups in these models

^b^The intercept of Item 2 was freed to vary between groups in these models

Confirming previous findings [8], we found moderate positive associations between health prevention focus and impaired mental health, *r*(2467) = .264, *p* < .001. Expanding on this, anxiety and depression exhibited comparable correlations, *r*(2467) = .246, *r*(2467) = .250, both *p* < .001. In contrast – again in line with [8] -, there were near-zero correlations for health promotion focus, *r*_Anxiety_(2467) = .059, *p* = .003, *r*_Depression_(2467) = .018, *p* = .371, *r*_PHQ-4_(2467) = .041, *p* = .042.

Norm values are reported in Table 4. Since there are no meaningful differences between the middle-aged (40–59 years) and the older (≥60 years) group, we merged these groups. We report means and standard deviations of the HRFS as well as quantiles to allow for the classification of individual scores.

**Table 4:** Norm values with quantiles

Gender	Female		Male	
Age (years)	≤39 (*n* = 449)	≥40 (*n* = 867)	≤39 (*n* = 411)	≥40 (*n* = 739)
HRFS promotion				
*M*	4.28	4.50	3.86	4.10
*SD*	1.44	1.37	1.48	1.49
1%	1.00	1.20	1.00	1.00
5%	1.40	2.00	1.00	1.40
10%	2.20	2.60	1.80	2.00
25%	3.40	3.60	2.80	3.00
50%	4.40	4.60	4.00	4.00
75%	5.20	5.40	4.80	5.20
90%	6.20	6.20	5.80	6.20
95%	6.40	7.00	6.40	6.60
99%	7.00	7.00	7.00	7.00
HRFS prevention				
*M*	3.16	3.56	2.78	3.33
* SD*	1.62	1.60	1.60	1.64
1%	1.00	1.00	1.00	1.00
5%	1.00	1.00	1.00	1.00
10%	1.00	1.50	1.00	1.00
25%	2.00	2.50	1.00	2.00
50%	3.00	3.50	2.50	3.50
75%	4.50	4.50	4.00	4.50
90%	5.50	6.00	5.00	5.50
95%	6.00	6.50	5.50	6.00
99%	7.00	7.00	7.00	7.00

## Discussion

The goal of the present study was to replicate and extend the findings by Schmalbach and colleagues [8] by testing the proposed HRFS model in a large sample representative of the German general population. This aim was accomplished. We found acceptable fit for the model proposed by Schmalbach and colleagues. Interestingly, the association between mental health and the health prevention focus was significantly smaller in the present study compared to the study by Schmalbach and colleagues [8]. This might be an effect of the skewed sample they used – over-representing young individuals. It is an indication that the correlation between the health regulatory focus and related constructs may differ across age.

The novel contribution of the present study lies in the analysis of measurement invariance, which previously had been lacking. Namely, we found the HRFS to be strictly invariant across gender, education, and family status, as well as partially strictly invariant across age groups and employment status. Additionally, we report norm values for age and gender groups (see Table 4). The findings of the present study allow researchers to apply the HRFS in a wider variety of research designs. Measurement invariance is an important prerequisite for comparisons between groups. Moreover, normative values will allow for the assessment of an individual’s health regulatory focus, which will be useful for designing health-focused interventions.

The HRFS is, as of now, the only reliable and valid tool available to German researchers for the assessment of the health regulatory focus. Ferrer, Lipkus, Cerully, McBride, Shepperd, and Klein [32] developed and tested an alternative version of an HRFS. However, they could not confirm their model in CFA.

## Limitations

Applications of the HRFS to address innovative research questions are still outstanding. As a reviewer pointed out, future research should look into how chronic diseases, migration status or cultural background impact health regulatory focus. These are important and innovative questions but were beyond the scope of the present article. Some research has already tackled similar issues [33, 34], but the health-specific HRFS will allow for large-scale surveys to answer these questions on a population level.

## Conclusion

The German HRFS was evaluated as a reliable and valid measure of the health regulatory focus. We have extended the findings by Schmalbach and colleagues [8] by adding an unambiguous analysis of measurement invariance and normative values. Thus, we can reiterate the original recommendation for the HRFS as a tool for psychological research into health behavior and motivation.

## Data Availability

The dataset used and analyzed in the current study is available from the corresponding author on reasonable request.

## Abbreviations

CFA: Confirmatory Factor Analysis
CFI: Comparative Fit Index
CMIN/DF: Minimum discrepancy divided by degrees of freedom
GH: Gamma hat
HRFS: Health Regulatory Focus Scale
PHQ: Patient Health Questionnaire
RMSEA: Root Mean Square Error of Approximation
SRMR: Standardized Root Mean Square Residual
TLI: Tucker-Lewis Index
USUMA: Unabhängiger Service für Umfragen, Methoden und Analysen [Independent Service for Surveys, Methods, and Analyses]
